# Supplementary material for: Profiling the expression and function of oestrogen receptor isoform ER46 in human endometrial tissues and uterine natural killer cells
Source: Hum Reprod. 2020 Feb 28;35(3):641–51. doi: 10.1093/humrep/dez306 (PMC7105323; doi:10.1093/humrep/dez306)
Supplement: SuppF1_dez306 [file suppf1_dez306.pdf]

**Human Reproduction, pp. 1–1, 2020**
doi:10.1093/humrep/dez306

human reproduction

# SUPPLEMENTARY DATA

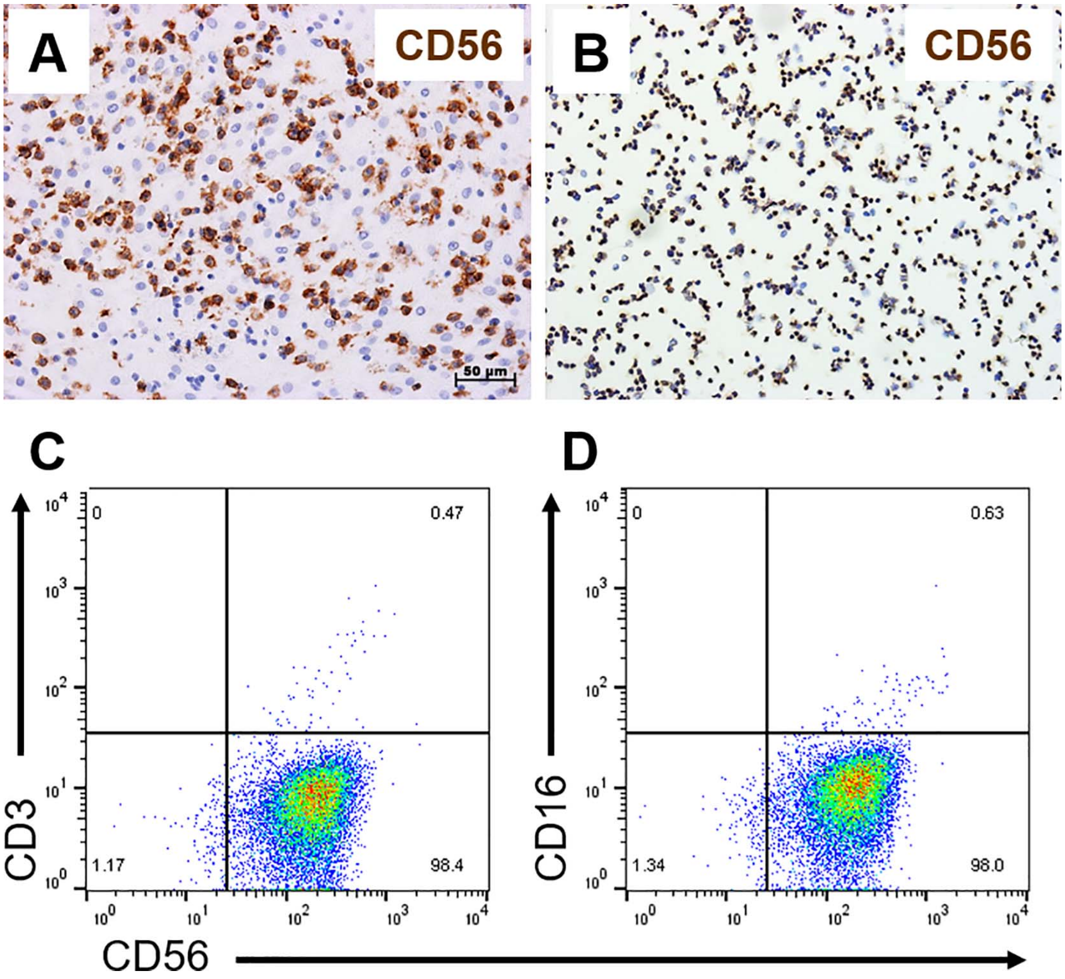


**Supplementary Figure S1 Characterisation and isolation of uNK cells from human first trimester decidua. (A)** Immunohistochemistry of primary human decidua demonstrating abundant expression of the uterine natural killer (uNK) cell marker CD56 (brown) throughout the tissue visualised using 3,3′-diaminobenzidine (DAB) chromogen (scale bar 50 µm). **(B)** Immunocytochemistry of isolated uNK cells expressing CD56 (brown) visualised using DAB chromogen (×10 magnification). Purity of isolated uNK cells was assessed by flow cytometry, and phenotype was confirmed as $CD56^+CD16^-CD3^-$. >98% of isolated cells were **(C)** $CD56^+CD3^-$ and **(D)** $CD56^+CD16^-$.
