# Supplementary figures and images for: Profiling the expression and function of oestrogen receptor isoform ER46 in human endometrial tissues and uterine natural killer cells
Source: Hum Reprod. 2020 Feb 28;35(3):641–51. doi: 10.1093/humrep/dez306 (PMC7105323; doi:10.1093/humrep/dez306)

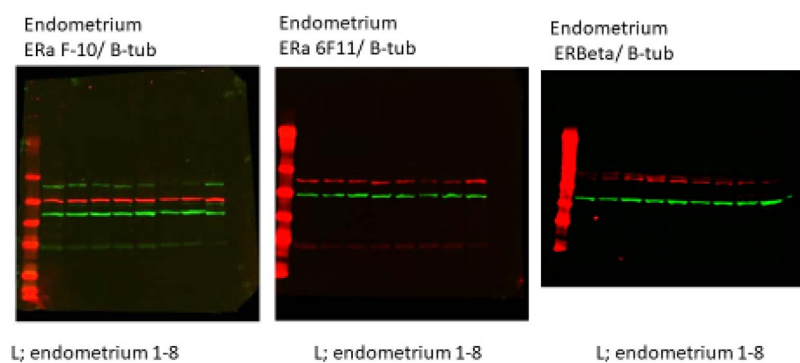

**Supplementary Figure S2** Uncropped western blot gel data, human endometrium.

Supplement: SuppF2_dez306 [file suppf2_dez306.pdf]

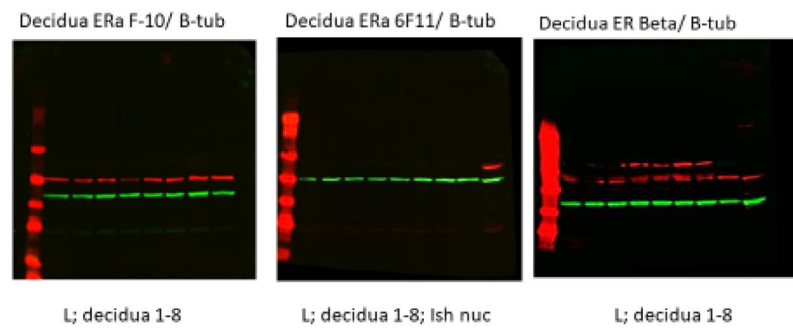

**Supplementary Figure S3** Uncropped western blot gel data, human first trimester decidua.

Supplement: SuppF3_dez306 [file suppf3_dez306.pdf]

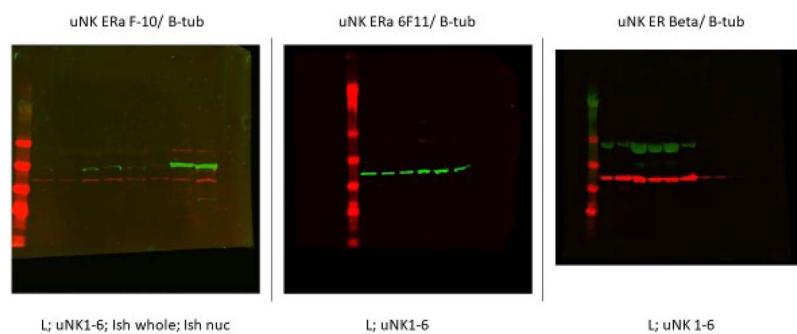

**Supplementary Figure S4** Uncropped western blot gel data, isolated uNK cells.

Supplement: SuppF4_dez306 [file suppf4_dez306.pdf]
