# Supplementary material for: Profiling the expression and function of oestrogen receptor isoform ER46 in human endometrial tissues and uterine natural killer cells
Source: Hum Reprod. 2020 Feb 28;35(3):641–51. doi: 10.1093/humrep/dez306 (PMC7105323; doi:10.1093/humrep/dez306)
Supplement: SuppF5_dez306 [file suppf5_dez306.pdf]

Human Reproduction, pp. 1–1, 2020
doi:10.1093/humrep/dez306

human reproduction

SUPPLEMENTARY DATA

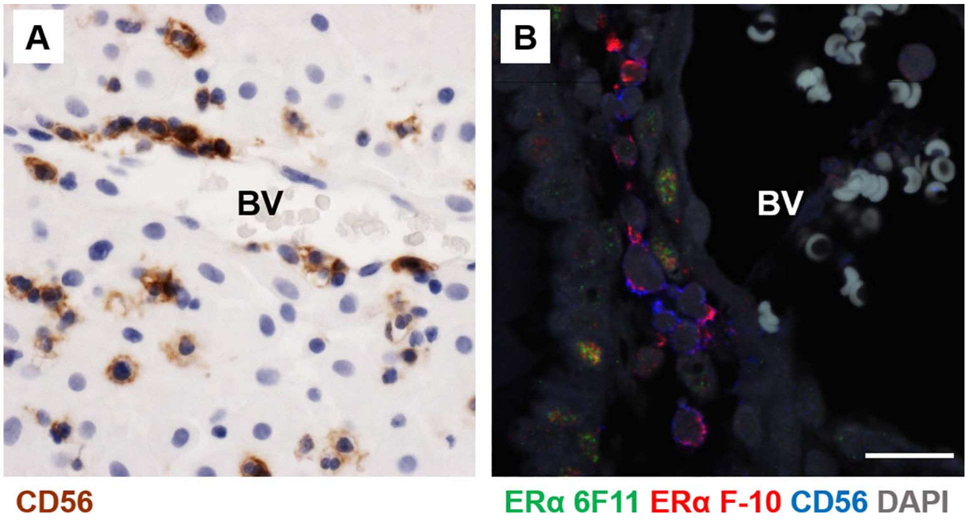

Supplementary Figure SV Immunohistochemistry demonstrating association of uNK with blood vessels in human first trimester decidua. (A) Immunohistochemistry of primary human decidua demonstrating accumulation of CD56-positive (brown) the uNK cells in peri-vascular regions of the tissue visualised using DAB chromogen (×40 magnification). **(B)** Multiplex immunohistochemistry using ERα 6F11, ERα F-10 and CD56 demonstrating accumulation of CD56-positive, ER46-positive uNK cells in perivascular region of human first trimester decidua (scale bar 20 μm). BV-blood vessel.
