# Supplementary material for: Profiling the expression and function of oestrogen receptor isoform ER46 in human endometrial tissues and uterine natural killer cells
Source: Hum Reprod. 2020 Feb 28;35(3):641–51. doi: 10.1093/humrep/dez306 (PMC7105323; doi:10.1093/humrep/dez306)
Supplement: SuppT1_dez306 [file suppt1_dez306.pdf]

Human Reproduction, pp. 1–1, 2020
doi:10.1093/humrep/dez306

human reproduction

SUPPLEMENTARY DATA

Supplementary Table SI Table of oligonucleotide sequences used in quantitative PCR analysis of human endometrium, first trimester decidua and isolated uNK cells.

Primer name	Accession number	Sequence	Primer position	UPL Probe
ESR1 (N-terminal) Forward	Nm_000125	*AACCAGTGCAACCATTGATAAAA*	1035–1056	69
ESR1 (N-terminal) Reverse	Nm_000125	*TCCTCTTCGGTCTTTTCGTATC*	1124–1145	69
ESR1 (C-terminal) Forward	Nm_001122741.1	*TCTGGAAAGACGTTCTTGATCC*	121–146	29
ESR1 (C-terminal) Reverse	Nm_001122741.1	*GGAGGGTCATGGTCATGGT*	216–234	29
ESR2 Forward	Nm_001437	*GCTCCTGTCCCACGTCAG*	1848–1865	62
ESR2 Reverse	Nm_001437	*TGGGCATTCAGCATCTCC*	1944–1961	62

uNK: uterine natural killer
