# Supplementary material for: Profiling the expression and function of oestrogen receptor isoform ER46 in human endometrial tissues and uterine natural killer cells
Source: Hum Reprod. 2020 Feb 28;35(3):641–51. doi: 10.1093/humrep/dez306 (PMC7105323; doi:10.1093/humrep/dez306)
Supplement: SuppT2_dez306 [file suppt2_dez306.pdf]

Human Reproduction, pp. 1–1, 2020
doi:10.1093/humrep/dez306

human reproduction

SUPPLEMENTARY DATA

Supplementary Table SII Primary antibodies used in immunohistochemistry and western blot analysis of human endometrium, first trimester decidua and isolated uNK cells.

Antibody name	Species	Supplier, catalogue no.
ERα (6F11)	Mouse	Vector, VP-614
ERα (F-10)	Mouse	Santa Cruz Biotechnology, sc-8002
ERβ (H-150)	Rabbit	Santa Cruz Biotechnology, sc-8974
NCAM (CD56)	Mouse	Invitrogen, 18–0152
β-Actin	Rabbit	Abcam, ab25894
β-Actin	Mouse	Sigma, A5441
β-Tubulin (H-235)	Rabbit	Santa Cruz Biotechnology, sc-9104
β-Tubulin (AC-15)	Mouse	Sigma-Aldrich, T4026

ER: oestrogen receptor, NCAM: neural cell adhesion molecule
