# Supplementary material for: Profiling the expression and function of oestrogen receptor isoform ER46 in human endometrial tissues and uterine natural killer cells
Source: Hum Reprod. 2020 Feb 28;35(3):641–51. doi: 10.1093/humrep/dez306 (PMC7105323; doi:10.1093/humrep/dez306)
Supplement: SuppT3_dez306 [file suppt3_dez306.pdf]

Human Reproduction, pp. 1–1, 2020
doi:10.1093/humrep/dez306

human reproduction

SUPPLEMENTARY DATA

Supplementary Table SIII Secondary antibodies used in immunohistochemistry and western blot analysis of human endometrium, first-trimester decidua and isolated uNK cells.

Antibody name	Species	Supplier, catalogue no.	Dilution
Anti-rabbit biotinylated	Goat	Vector Laboratories, BA-1000	1:500
Anti-mouse IgG biotinylated	Goat	Vector Laboratories, BA-9200	1:500
Anti-mouse Peroxidase	Goat	Dako, P0447	1:200
IRDye 680 RD Anti-mouse	Donkey	Licor, 926-68072	1:10000
IRDye 680 RD Anti-rabbit	Donkey	Licor, 926-68073	1:10000
IRDye 800 CW Anti-mouse	Donkey	Licor, 32212	1:10000
IRDye 800 CW Anti-rabbit	Donkey	Licor, 926-32213	1:10000
Strepavidin (horseradish peroxidase)	N/A	Vector Laboratories, SA-5004	1:500
