# Supplementary material for: Profiling the expression and function of oestrogen receptor isoform ER46 in human endometrial tissues and uterine natural killer cells
Source: Hum Reprod. 2020 Feb 28;35(3):641–51. doi: 10.1093/humrep/dez306 (PMC7105323; doi:10.1093/humrep/dez306)
Supplement: SuppT4_dez306 [file suppt4_dez306.pdf]

Human Reproduction, pp. 1–1, 2020
doi:10.1093/humrep/dez306

human reproduction

SUPPLEMENTARY DATA

Supplementary Table SIV ER66 western blot densitometry; human endometrium.

Channel	Lane and band	Signal	densitometry (ER66/tubulin)
R	1 ER66 prolif endo	14 600	1.035460993
G	1 tubulin prolif endo	14 100	
R	2 ER66 prolif endo	12 500	0.925925926
G	2 tubulin prolif endo	13 500	
R	3 ER66 prolif endo	10 600	0.560846561
G	3 tubulin prolif endo	18 900	
R	4 ER66 prolif endo	12 900	0.777108434
G	4 tubulin prolif endo	16 600	
R	5 ER66 secretory endo	10 300	0.735714286
G	5 tubulin secretory endo	14 000	
R	6 ER66 secretory endo	5280	0.502857143
G	6 tubulin secretory endo	10 500	
R	7 ER66 secretory endo	7990	0.505696203
G	7 tubulin secretory endo	15 800	
R	8 ER66 secretory endo	20 200	1.224242424
G	8 tubulin secretory endo	16 500	
