# Supplementary material for: Profiling the expression and function of oestrogen receptor isoform ER46 in human endometrial tissues and uterine natural killer cells
Source: Hum Reprod. 2020 Feb 28;35(3):641–51. doi: 10.1093/humrep/dez306 (PMC7105323; doi:10.1093/humrep/dez306)
Supplement: SuppT5_dez306 [file suppt5_dez306.pdf]

Human Reproduction, pp. 1–1, 2020
doi:10.1093/humrep/dez306

human reproduction

SUPPLEMENTARY DATA

Supplementary Table SV **ER46 western blot densitometry; human endometrium.**

Channel	Lane and band	Signal	densitometry (ER46/tubulin)
G	1 ER46 prolif endo	18 600	0.801724138
R	1 tubulin proli endo	23 200	
G	2 ER46 prolif endo	17 600	0.611111111
R	2 tubulin proli endo	28 800	
G	3 ER46 prolif endo	21 700	0.818867925
R	3 tubulin proli endo	26 500	
G	4 ER46 prolif endo	17 100	0.564356436
R	4 tubulin proli endo	30 300	
G	5 ER46 secretory endo	25 500	0.84717608
R	5 tubulin secretory endo	30 100	
G	6 ER46 secretory endo	13 400	0.748603352
R	6 tubulin secretory endo	17 900	
G	7 ER46 secretory endo	21 100	1.024271845
R	7 tubulin secretory endo	20 600	
G	8 ER46 secretory endo	17 300	0.483240223
R	8 tubulin secretory endo	35 800	
