# Supplementary material for: Profiling the expression and function of oestrogen receptor isoform ER46 in human endometrial tissues and uterine natural killer cells
Source: Hum Reprod. 2020 Feb 28;35(3):641–51. doi: 10.1093/humrep/dez306 (PMC7105323; doi:10.1093/humrep/dez306)
Supplement: SuppT6_dez306 [file suppt6_dez306.pdf]

Human Reproduction, pp. 1–1, 2020
doi:10.1093/humrep/dez306

human reproduction

SUPPLEMENTARY DATA

Supplementary Table SVI ERβ western blot densitometry; human endometrium.

Channel	Lane and band	Signal	densitometry (ERβ/actin)
R	1 ERβ prolif endo	196 000	0.388888889
G	1 actin prolif endo	504 000	
R	2 ERβ prolif endo	280 000	0.710659898
G	2 actin prolif endo	394 000	
R	3 ERβ prolif endo	508 000	0.675531915
G	3 actin prolif endo	752 000	
R	4 ERβ prolif endo	377 000	0.646655232
G	4 actin prolif endo	583 000	
R	5 ERβ secretory endo	449 000	0.626220363
G	5 actin secret endo	717 000	
R	6 ERβ secretory endo	334 000	0.713675214
G	6 actin secret endo	468 000	
R	7 ERβ secretory endo	369 000	0.566820276
G	7 actin secret endo	651 000	
R	8 ERβ secretory endo	266 000	0.405487805
G	8 actin secret endo	656 000	
