# Supplementary material for: Profiling the expression and function of oestrogen receptor isoform ER46 in human endometrial tissues and uterine natural killer cells
Source: Hum Reprod. 2020 Feb 28;35(3):641–51. doi: 10.1093/humrep/dez306 (PMC7105323; doi:10.1093/humrep/dez306)
Supplement: SuppT7_dez306 [file suppt7_dez306.pdf]

Human Reproduction, pp. 1–1, 2020
doi:10.1093/humrep/dez306

human reproduction

SUPPLEMENTARY DATA

Supplementary Table SVII ER66 western blot densitometry; human first trimester decidua.

Channel	Lane and band	Signal	densitometry (ER66/tubulin)
G	1 tubulin decidua	11 400	0.080965
R	1 ER66 decidua	923	
G	2 tubulin decidua	17 600	0.014148
R	2 ER66 decidua	249	
G	3 tubulin decidua	15 600	0.00891
R	3 ER66 decidua	139	
G	4 tubulin decidua	15 100	0.028477
R	4 ER66 decidua	430	
G	5 tubulin decidua	15 700	0.021847
R	5 ER66 decidua	343	
G	6 tubulin decidua	20 600	0.03767
R	6 ER66 decidua	776	
G	7 tubulin decidua	23 800	0.035672
R	7 ER66 decidua	849	
G	8 tubulin decidua	23 100	0.001775
R	8 ER66 decidua	41	
