# Supplementary material for: Profiling the expression and function of oestrogen receptor isoform ER46 in human endometrial tissues and uterine natural killer cells
Source: Hum Reprod. 2020 Feb 28;35(3):641–51. doi: 10.1093/humrep/dez306 (PMC7105323; doi:10.1093/humrep/dez306)
Supplement: SuppT8_dez306 [file suppt8_dez306.pdf]

**Human Reproduction, pp. 1–1, 2020**
doi:10.1093/humrep/dez306

human reproduction

# SUPPLEMENTARY DATA

**Supplementary Table SVIII** **ER46 western blot densitometry; human first trimester decidua.**

| Channel | Lane and band | Signal | densitometry (ER46/tubulin) |
|---|---|---|---|
| R | 1 tubulin decidua | 17 600 | 0.880681818 |
| G | 1 ER46 decidua | 15 500 | |
| R | 2 tubulin decidua | 12 300 | 1.138211382 |
| G | 2 ER46 decidua | 14 000 | |
| R | 3 tubulin decidua | 14 700 | 1.238095238 |
| G | 3 ER46 decidua | 18 200 | |
| R | 4 tubulin decidua | 8380 | 2.136038186 |
| G | 4 ER46 decidua | 17 900 | |
| R | 5 tubulin decidua | 16 900 | 0.98816568 |
| G | 5 ER46 decidua | 16 700 | |
| R | 6 tubulin decidua | 17 300 | 1.057803468 |
| G | 6 ER46 decidua | 18 300 | |
| R | 7 tubulin decidua | 25 000 | 1.068 |
| G | 7 ER46 decidua | 26 700 | |
| R | 8 tubulin decidua | 32 400 | 0.632716049 |
| G | 8 ER46 decidua | 20 500 | |
