# Supplementary material for: Profiling the expression and function of oestrogen receptor isoform ER46 in human endometrial tissues and uterine natural killer cells
Source: Hum Reprod. 2020 Feb 28;35(3):641–51. doi: 10.1093/humrep/dez306 (PMC7105323; doi:10.1093/humrep/dez306)
Supplement: SuppT9_dez306 [file suppt9_dez306.pdf]

Human Reproduction, pp. 1–1, 2020
doi:10.1093/humrep/dez306

human reproduction

SUPPLEMENTARY DATA

Supplementary Table SIX **ERβ western blot densitometry; human first-trimester decidua.**

Channel	Lane and band	Signal	densitometry (ERβ/actin)
R	1 ERβ decidua	16 700	0.566102
G	1 actin decidua	29 500	
R	2 ERβ decidua	11 100	0.327434
G	2 actin decidua	33 900	
R	3 ERβ decidua	17 700	0.487603
G	3 actin decidua	36 300	
R	4 ERβ decidua	19 200	0.474074
G	4 actin decidua	40 500	
R	5 ERβ decidua	18 500	0.420455
G	5 actin decidua	44 000	
R	6 ERβ decidua	23 300	0.605195
G	6 actin decidua	38 500	
R	7 ERβ decidua	20 900	0.48046
G	7 actin decidua	43 500	
R	8 ERβ decidua	37 000	1.091445
G	8 actin decidua	33 900	
